# Ontology for Genome Comparison and Genomic Rearrangements

**DOI:** 10.1002/cfg.436

**Published:** 2004

**Authors:** Keith Flanagan, Robert Stevens, Matthew Pocock, Pete Lee, Anil Wipat

**Affiliations:** 1 School of Computing Science University of Newcastle upon Tyne Claremont Tower Claremont Road NE1 7RU UK; 2 Department of Computing Science University of Manchester UK

## Abstract

We present an ontology for describing genomes, genome comparisons, their evolution and biological function. This ontology will support the development of novel genome comparison algorithms and aid the community in discussing genomic evolution. It provides a framework for communication about comparative genomics, and a basis upon which further automated analysis can be built. The nomenclature defined by the ontology will foster clearer communication between biologists, and also standardize terms used by data publishers in the results of analysis programs. The overriding aim of this ontology is the facilitation of consistent annotation of genomes through computational methods, rather than human annotators. To this end, the ontology includes definitions that support computer analysis and automated transfer of annotations between genomes, rather than relying upon human mediation.


**Comparative and Functional Genomics**
*Comp Funct Genom* 2004; **5**: 537–544.
Published online in Wiley InterScience (www.interscience.wiley.com). **DOI:** 10.1002/cfg.436

**Conference Paper**

### Ontology for genome comparison and genomic rearrangements

Keith Flanagan,[1] Robert Stevens[2], Matthew Pocock[1], Pete Lee[1] and Anil Wipat[1]*
[1] *School of Computing Science, University of Newcastle upon Tyne, UK*
[2] *Department of Computing Science, University of Manchester, UK*

**Correspondence to:*
*Anil Wipat, School of Computing Science, Claremont Tower, Claremont Road, Newcastle upon Tyne NE1 7RU, UK.*
*E-mail: anil.wipat@ncl.ac.uk*

Received: 25 October 2004
Revised: 27 October 2004
Accepted: 1 November 2004

### Abstract

**We present an ontology for describing genomes, genome comparisons, their evolution and biological function. This ontology will support the development of novel genome comparison algorithms and aid the community in discussing genomic evolution. It provides a framework for communication about comparative genomics, and a basis upon which further automated analysis can be built. The nomenclature defined by the ontology will foster clearer communication between biologists, and also standardize terms used by data publishers in the results of analysis programs. The overriding aim of this ontology is the facilitation of consistent annotation of genomes through computational methods, rather than human annotators. To this end, the ontology includes definitions that support computer analysis and automated transfer of annotations between genomes, rather than relying upon human mediation.**
**Copyright © 2004 John Wiley & Sons, Ltd.**

**Keywords:** **comparative; genome; logic; ontology; rearrangement; reasoning**

### Introduction

The sequencing of whole genomes is becoming more cost-effective as developments in high-throughput technology continue to advance. For microbial genomes in particular, the acquisition of a complete genome sequence is almost becoming routine, providing a platform on which to base more advanced biological investigations. Consequently, at the time of writing, over 200 bacterial genomes have already been completed, and many more are planned.

One of the major aims of the molecular biologist is to get a better understanding of an organism's phenotype from its genomic sequence. Whilst biological knowledge can be gained by algorithmic analysis of the genome sequence itself (Delcher *et al.*, 1999b; Kurtz and Schleiermacher, 1999; Wexler *et al.*, 2004), the comparative analysis of genomes is one of the most effective ways of extracting knowledge about the genetic basis for an organism's phenotype (Parkhill, 2002) and the goal of this work is to add semantic support to such analyses through the use of an ontology.

As the size of sequence databases increases, computational approaches become increasingly desirable. However, methods to derive knowledge and hypotheses from comparative genome analyses are currently still relatively limited. Routine comparative genome analyses are essentially based on the use of tools which establish sequence similarity at the nucleotide or protein sequence level. From this information it is possible to establish areas of synteny between genomic regions and to discover potentially homologous coding regions. Implementations of algorithms such as Blast (Altschul *et al.*, 1990), Fasta (Pearson and Lipman, 1988), Smith-Waterman (Smith & Waterman, 1981) and Mummer (Delcher *et al.*, 1999a) are typically used in an all-against-all fashion to generate a baseline of computationally assigned comparative data. Whilst the information regarding potentially homologous coding regions may be used to assign putative function and assign gene families, information

Copyright © 2004 John Wiley & Sons, Ltd.

regarding sequence synteny between genome data does not commonly appear as primary sequence annotation and is regarded by many as 'secondary' genomic information.

This paper describes an ontology that we hope will enable the development of advanced algorithms for the comparison and analysis of entire genomes, primarily from prokaryotic organisms. Automated genome comparison is a complex task, and thus requires formal descriptions and semantics of domain terms to be defined in order to enable logical reasoning. The algorithms facilitated by this ontology will address the need for increased throughput in the area of comparative genomics.

### Automated genome analysis and comparison

Traditional approaches to comparative analysis of genomes involve creating links between primary genomic information, e.g. genomic regions or features, within a single genome or across genomes and species. In a similar fashion to genomic sequence annotation, the assignment of these links may be done by human expert curation or using computational approaches.

The derivation of biological knowledge from basic computational comparative analyses currently relies heavily on strategies for human annotation. Standard approaches have utilised visualization based tools such as Pipmaker (Schwartz *et al.*, 2000) and ACT (Artemis Comparison Tool) [**http://www.sanger.ac.uk/Software/ACT/** (ACT, accessed August 2004)], in combination with computationally generated comparative data and, in the case of higher eukaryotes, experimentally derived linkage data, to guide the scientist in the generation of hypotheses about more complex relationships between genomic regions and the products they encode. These relationships include evolutionary relationships, such as homology, orthology, paralogy, physical relationships, genomic rearrangements and relationships derived by lateral gene transfer. This approach has been shown to be very valuable for deriving knowledge from focused studies on finite genomic regions, for a limited number of genomes (Anjum *et al.*, 2003).

However, visualization-based approaches are limited by the analytical capacity of the human user and do not scale well for large numbers of complete genomic sequences. Further automated analysis techniques must be developed to allow us to deal with the influx of new genome sequences, moving some of the more mundane analysis currently performed by the biologist into the computational domain. This analysis must take the form of both the generation of comparative links between genomic regions and the traversal of those links between genomes for evolutionary and functional knowledge generation.

### Ontology-backed algorithmic approach

One major limitation in the development of computational approaches to deriving knowledge from comparative genomics is the lack of standards for terminology. Whilst most biological communities have developed nomenclatures, many are specific to a particular area of study. It is common to see different terms used to represent similar concepts, similar terms used to represent different concepts, and terms with shared meanings between communities. Recently, a number of initiatives have been established in an attempt to standardize the use of terms in molecular biology and genomics. The most relevant of these to comparative genomics are the Gene Ontology (GO) (The Gene Ontology Consortium, 2000) and Sequence Ontology (SO) [**http://song.sourceforge.net** (Sequence Ontology, accessed August 2004)] projects. The GO project is an effort to address the need for consistent descriptions of gene products in different databases, e.g. Mouse Genome Informatics [**http://www.informatics.jax.org/** (Mouse Genome Informatics, accessed August 2004)] and includes standard terms that fall into the categories of molecular function, biological process and cellular components. The SO project is part of the GO project and aims to develop an ontology and software modules to be used to describe and communicate biological sequence information. In practice, SO comprises a set of terms that may be used to semantically mark up a nucleotide sequence with features that describe it. It includes what the developers refer to as 'raw' features, e.g. nucleotide similarity hits, and also interpretations, e.g. models for genes. SO facilitates comparative genomics approaches by providing a standard set of terms for referring to the concepts of sequence features. Comparisons between genomes are simplified if both genomes have been marked

Copyright © 2004 John Wiley & Sons, Ltd. 

up with standard terms that define how an individual feature can be recognized to belong to that concept or feature. For example, the use of SO allows features annotated on one genome to be compared against those on another genome, providing information about the conservation of gene structure, and permitting the transfer of equivalent annotations. It also permits areas of sequence similarity between genomes to be marked up and includes terms for the description of mutations and chromosomal rearrangements.

Currently, SO stops short of defining a complete set of terms for describing evolutionary events that are amenable to automated computational analysis. The development of novel computational approaches for comparing genomes and deriving knowledge from those comparisons requires a standard set of terms for comparative genomics. This set of terms may take the form of an ontology which spans biological research niches, and is then mapped down to individual domains or an agreed common standard ontology for all biological species. The establishment of such an ontology will facilitate computational assignment of 'secondary' annotation, i.e. comparative genomic annotations. This, in turn, will allow the development of computational approaches for analysing, comparing and inferring annotations between genomes.

The motivation behind developing the ontology described in this work is two-fold. First, in order to perform computational analyses of genomic comparison data in a rigorous fashion, it is necessary to have a set of formally defined concepts to reason about. These concepts must be enriched with unambiguous relations to enable logical reasoning and inference to be performed over data annotated with the concepts. Second, it is important to be able to share the annotated information with other biologists and also other software tools.

The rationale behind the production of this ontology is to develop a system that will fully support automated computational approaches for logical reasoning over genomic sequence data and annotations. Currently available approaches are limited in their application to this domain, since they tend to place an emphasis on human-based interpretation and assignment. Our application domain requires the ability to incorporate probabilistic and machine learning strategies into the methods for assigning annotations, primarily through genomic comparison. We require functionality that will facilitate the integration of relationships between entities derived from sequence similarity with those derived through prior knowledge of evolutionary, functional and phenotypic traits.

One particularly valuable benefit of this approach that is of interest to our research, is the automatic discovery and annotation of the contribution that genomic rearrangements may make to an organism's phenotype. For example, by employing such a strategy it will be possible to infer the functional contribution of an inserted sequence in a probabilistic way from a prior consideration of its possible evolutionary origins, together with the putative function of the genes found on the fragment. Other types of rearrangement events, such as inversions, repeats and translocations, may also be analysed in a similar fashion. Thus, the ultimate goal of this work is to assemble the ontological framework that will facilitate machine-based approaches to performing these analyses at a cross-genomic scale.

In this paper we describe the framework for an ontology that will enable such a scenario and this ontology will initially focus on prokaryotic genomes. We will build on the work carried out in the GO and SO projects with a clear focus of promoting computational-based comparative genomic analysis.

### Materials and methods

We are developing ontologies using OWL-DL (Web Ontology Language-Description Logic; McGuinness and van Harmelen, 2004) and the reasoner RACER (Reasoner for A-Boxes and Concept Expressions Renamed; Haarslev and Möller, 2001) within Protégé (Gennari *et al*., 2002). OWL-DL was chosen as it is emerging as a standard for representing ontologies amenable to formal reasoning methods. RACER adds to OWL-DL some capabilities, such as the ability to test for consistency and infer classifications based upon formal, explicit, computationally amenable descriptions of classes. Protégé is a free, open-source editor for Semantic Web applications, including support for RDF, OWL-DL and RACER. Protégé can be configured for community-based curation and allows custom viewers and editors to be integrated, using a plug-in API that could be used to provide bioinformatics-specific capabilities.

Copyright © 2004 John Wiley & Sons, Ltd. 

The terms used for classes that form the ontologies are being collected using two strategies. First, we are adding those terms and relationships that are required for modelling comparative genomics databases, such as Microbase [**http://www.microbase.org.uk** (Microbase, accessed September 2004)]. Second, we are requesting input from the comparative genomics community to acquire terms and relationships that are relevant to the wider community.

### Results

The classes in our ontology have been split into seven sub-domains, or layers. There are several reasons for this. First, it is desirable to arrange concepts into logical modules, allowing multiple levels of abstraction, while reducing complexity at each level (Devedzic, 2002). This is analogous to how modern programming languages allow code to be grouped into modules or packages. The user need not import terms from a level outside his/her scope of interest. For example, if a user is only interested in performing pair-wise comparisons, he/she need not concern him/herself with terms dealing with evolutionary history. Furthermore, a layered ontology is a more modular ontology, with clear divisions between the purpose each layer serves, and by splitting definitions into their respective topics, it is easier to see where each sub-domain links to the others. It is hoped that this approach will facilitate the re-use of individual domains within other projects.

In addition to providing separation between topics, the sub-domains have different primary purposes. The rationale behind the ontology is to allow the automated comparison and analysis of biological sequences. This imposes two requirements: storage and reasoning. Therefore, some domains are designed such that data structures such as class hierarchies (in the programming sense) and methods, may be derived from the classes in the ontology. The remaining sub-domains contain terms used primarily for reasoning over the stored data, i.e. the higher level concepts that may or may not occur in a particular data set. The reasoning sub-domains add biological meaning to the raw sequence and rearrangement data. We propose the following sub-domains:

1. *Physical components* — provides a basic set of terms for describing physical entities, and their compositions.
2. *Single sequence* — allows regions of a genome sequence to be addressed, and marked as 'of interest'.
3. *Biological annotation of single sequences* — annotates a region of sequence with biological meaning.
4. *Pair-wise comparison* — describes the physical rearrangements (similarities and differences) between two genome sequences.
5. *Biological annotation of pair-wise comparisons* — describes the biological consequences of the physical rearrangement events.
6. *Evolutionary history* — provides terms for describing how a set of sequences are related to eachother.
7. *Biological annotation of evolutionary history* — provides terms for annotating evolutionary histories with biological implications.

Much consideration has gone into the possibility of re-using terms from existing ontologies, such as GO and SO. We have identified the areas where our ontology shares common ground with both of these ontologies. However, the GO and SO projects remain primarily focused on computer-aided human annotation of biological sequences, whereas the goal for our ontology is enabling human-aided machine annotation of these sequences. While our ontology is intended to operate within some of the domains covered by GO and SO, it has a different primary purpose. Our ontology is required to have many formally defined constraints and relations. This makes a direct use of terms in the SO/GO ontologies impossible; however, it should be feasible to link from our terms to relevant terms defined in other bioinformatics ontologies, where suitable candidates exist. This will be done wherever possible.

The areas most likely to overlap with the GO/SO ontologies are the first three sub-domains of our ontology, e.g. the 'Physical components' sub-domain has similar terms to those defined in the Gene Ontology, while the sub-domains dealing with single sequence have counterparts in the Sequence Ontology.

Copyright © 2004 John Wiley & Sons, Ltd.

### Physical components

The physical components sub-domain contains definitions of physical entities related to genome structure and containment, e.g. this domain includes terms such as 'cell', 'chromosome', 'nucleus', and 'plasmid'. While not intending to be an exhaustive set of physical entity definitions, this sub-domain contains definitions of the physical entities referenced by concepts in the higher layers. It is important that a given ontology should concern itself only with the domain it represents. The physical component sub-domain defines the boundary of our ontology. It is for this reason that the physical component sub-domain is little more than a dictionary of terms, with simple relations and few constraints, analogous to a black-box system. For example, we might have a definition 'sequence' in the 'single sequence' sub-domain that states that the physical representation of a sequence may be in the form of either a chromosome or a plasmid. It is also useful to state that plasmids are mobile genetic elements, whereas a chromosome is static. However, there is no need (for our purposes) to go into great detail for many of the terms, e.g. it is not necessary to define exactly which processes are involved in the conjugative transfer of a plasmid from one organism to another; we only need to know that it can move.

The primary purpose of this sub-domain is to provide some basic terms on which the higher level sub-domains depend. If more detailed definitions are required in the future, it should be possible to link terms with third-party ontologies that have a greater focus on the area in question.

### Single sequence

The terms in this sub-domain relate to identifiable points of interest that occur within a single sequence. By way of example, we define a 'sequence' as being composed of one or two 'strands'. A 'strand' consists of a string of nucleotides. We describe a 'region' as being an area of a 'strand' with a particular starting point and length. From this we can build the definitions of 'base', 'base-pair', and so on. A range of relationships between these concepts are also defined, allowing concepts such as 'complementary' and 'reversal' of sequences to be unambiguously defined. The single sequence domain also allows relative ordering of regions to be described, allowing grammars over regions to be constructed. The aim of this sub-domain is to provide the essential framework of terms upon which biological meaning can be applied. The terms defined here will be used primarily for constructing data hierarchies for storing biological data for later analysis. Libraries of useful functions will be developed to manipulate data defined using terms from this sub-domain. In this respect, the purpose of the definitions in this sub-domain is similar to definitions of terms that are already employed by projects providing programming libraries for sequence analysis, such as BioJava [**http://www.biojava.org** (BioJava, accessed August 2004)], BioPerl (Stajich *et al.*, 2002) and BioPython (Chapman and Chang, 2000). Terms for this sub-domain are being drawn from these projects and from the Sequence Ontology wherever possible.

### Biological annotation of single sequences

The terms defined in the 'single sequence' sub-domain give us the ability to locate regions of interest within a genome sequence of interest, on a particular strand. The next stage is to be able to annotate these regions with biological knowledge. This sub-domain provides us with mechanisms to associate terms of classes for entities such as 'operon', 'gene', 'promoter', 'terminator', 'Shine–Dalgarno site', and so on with a particular region of interest. We are then able to build up a library of archetypes (well-defined patterns of concepts that occur in a particular order) relating to a single sequence from the terms defined in the single sequence sub-domain.

The example shown in Figure 1 is an adaptation of a 'correia element' (Parkhill *et al.*, 2000, Figure 2). The correia element is a sequence flanked by inverted repeats, although the terminating repeat is optional. A deletion may also be present within the region shown. One of the major aims of our work is to promote the computational assignment of such archetypes. Armed with the single sequence definitions, and the compound archetype definitions such as a correia element, it is now possible to develop algorithms to find instances of the archetypes in an automated fashion. We also aim to generate further compound definitions automatically, using pattern discovery algorithms.

Copyright © 2004 John Wiley & Sons, Ltd.

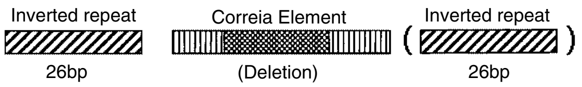

**Figure 1.** An example of a 'compound' definition (adapted from Parkhill *et al.*, 2000, Figure 2). It consists of an inverted repeat 26 bases in length, followed by a region of sequence that may or may not contain a deletion. Finally, there is an optional terminating inverted repeat. If an annotated genome contains the above pattern of terms, then they may be collectively named a 'correia element'

### Pair-wise comparison

The pair-wise comparison sub-domain introduces the concept of a source sequence and a target sequence. Terms are provided for describing the similarity and differences between two genome sequences. With these terms, and combined with appropriate detection algorithms, it is possible to build up an idea of how and where one sequence differs, or is similar to the other sequence. For instance, a deletion event is defined as 'a region occurring in the source genome that does not occur in the target genome, which is flanked by matching regions'.

Similarly, a general definition for a repeated element is: 'a region occurring in the source genome that has multiple occurrences in the target genome'. Using inheritance and logical constraints, it is possible to describe stricter definitions for particular a type of repeat. For example, a tandem repeat is a repeat whereby all the occurrences in the target genome have a distance between the end of one repeat element and the beginning of the next element that is lower than some threshold. We take the view that sequence edits are always taken to be with respect to the source sequence. For example, suppose the comparison of sequences S1 against S2 reveals a deletion. If the sequences had been compared in the order S2 against S1, the same edit would have been classified as an insertion. Changes always occur from the source sequence, to the target sequence. There is no concept in this sub-domain of temporal order; we are concerned simply with comparing two arbitrary sequences.

Given the rate at which microbial genomes mutate, the terms defined in this sub-domain lend themselves nicely to describing the rearrangement events between the genomes of these organisms. In the comparison of the *Escherichia coli* K-12 genome against several close relatives (McClelland *et al.*, 2000), a number of interesting genomic rearrangement events are described, including over 160 deletions from the genome *Escherichia coli* when compared against *Salmonella enterica* serovars Typhimurium, Typhi, and Paratyphi A. We consider that this type of query, 'Find regions absent in genome A that do occur in genomes B, C and D', a good candidate for automation. Performing such a query manually would require a large amount of work. Given genome sequence data, and the formal definitions for describing the various genome rearrangements, it is possible to develop algorithms for answering these kinds of queries in an automated fashion, allowing entire databases to be systematically searched.

### Biological annotation of pair-wise comparisons

Taking the terms of the 'pair-wise comparison' domain a step further, the 'biological annotation of pair-wise comparisons' domain adds biological meaning to sequence similarities and edits. For instance, a region annotated as a 'matching region' in the 'pair-wise comparison' sub-domain, may be annotated as a 'homologous' or 'syntenic' region in the 'biological annotation of pair-wise comparisons' sub-domain. In other words, the terms from the 'pair-wise comparison' domain indicate that a particular sequence region is similar between genomes without implying similar function. The 'biological annotation of pair-wise comparisons' sub-domain allows us to associate biological meaning to the similarities between sequences.

Applying biological meaning to regions of sequence enables us to maintain exhaustive provenance regarding the transfer of an annotation from one sequence to another. Maintaining detailed provenance is essential for verifying the accuracy of the classification decisions made by the computational methods.

### Evolutionary history

When performing pair-wise comparisons, we do not consider temporal ordering. However, once several pair-wise comparisons have been completed, it is be possible to infer temporal ordering of the mutation events involving a region of sequence. For instance, if a particular region of sequence is tracked over time, it may incur several insertions or deletions. The terms defined in this sub-domain

Copyright © 2004 John Wiley & Sons, Ltd. 

allow us to put these edits into context by arranging the sequences into tree structures.

The aim of this sub-domain is to provide terms and relations that allow the ordering of evolutionary events to be established. We define concepts and relations that can be used for building and manipulating trees. The 'evolutionary history' sub-domain contains formal definitions for concepts, such as 'root', 'internal' and 'leaf notes'. We can then define the relations between these types of node, such as 'parent', 'child' and 'sibling'. Using these terms we can describe both vertical and horizontal evolution, allowing us to put pair-wise rearrangements into context.

### Biological annotation of evolutionary histories

In much the same way as we need to apply biological meaning to pair-wise rearrangements, we must be able to assess the biological significance of the positions of sequences or subsequences in the context of structures that describe evolutionary histories. For example, given a gene duplication event within a single genome, we require terms that allow us to assert that a copy of a gene is a paralogue, and also allow us to state which genes in other sequences within the tree have associated paralogues.

### Discussion

In this paper we outline an ontology for annotating genomic comparisons and genomic rearrangements. An ontology provides us with a collection of concepts that we can reason about. However, given the current scale of the sequence databases, the assignment of these terms to annotate genomic comparisons and to reason about them requires computational methods. Our proposed computational approaches to knowledge derivation have requirements on the ontology that are distinct from a more human-orientated ontology, such as GO or SO. The ontology that we present therefore builds on existing ontologies, supplementing them with terms and structure to facilitate the computational use and assignment of ontology terms to biological data.

The ontology presented here is rich in relations and constraints. This is a very important aspect of the project, if we are to successfully develop automated systems capable of reasoning over biological data and transferring annotations from well-characterized genomic regions to unannotated regions. Using a description logic language, we are able to check the ontology terms for consistency, using an inference engine such as RACER. This ontology provides us with a set of logically sound and unambiguous descriptions of terms, and a framework on which data structures and algorithms can be built. Our ontology is currently under active development using OWL-DL (McGuinness and van Harmelen, 2004). OWL-DL on its own is insufficient for defining many of the constraints we require, specifically the ability to constrain over data values (Haarslev and Möller, 2003). In order to overcome these limitations, we are currently investigating the use of the RACER extensions, specifically its ability to reason over A-boxes (data in instance definitions).

The main area in which we contend that these ontology definitions will prove to be essential is in the research and development of various pattern-matching algorithms for genomic comparison. Utilizing the formal concept definitions and associated library functions, these algorithms will attempt to annotate sequence comparisons with concepts such as the rearrangement events that we have described in the pair-wise comparison sub-domain. Rather than performing a pair-wise comparison that simply generates more data, the biological knowledge encoded in the ontology concepts will be used to apply meaning to various regions of sequences. We are developing algorithms that will use a probabilistic approach of reaching conclusions based on the domain knowledge provided by the ontology, and based on the data available. Applying prior knowledge to current situations occurs very frequently in biology, and this makes it an ideal candidate for automated ontology-driven systems.

Another future aim of this work is to research pattern discovery algorithms to look for interesting aggregates of terms that occur in several genomes, or pair-wise comparisons of genomes. If found, these patterns may indicate a biologically significant archetype. It is hoped that we can use these algorithms to point out features for which there is not currently an ontology term.

As discussed earlier, the subject area of several of our sub-domains overlap with existing ontologies. For instance, the 'Biological annotation of

Copyright © 2004 John Wiley & Sons, Ltd.

single sequences' sub-domain overlaps quite heavily with the SO project. While every effort will be made to forge links with terms in other relevant bioinformatics ontologies, a direct one-to-one mapping between our terms and other ontologies is likely to be impossible, although it should be feasible to provide a translation. The ability to translate between our ontology terms and the terms of another ontology (where sufficient overlap exists, e.g. with SO) would enable comparisons between sequences marked up in either ontology to be made. This in turn would allow us to test the accuracy of our automatically generated annotations against those produced with human annotators, using the terms of the Sequence Ontology.

We hope that ultimately this ontology and the associated algorithms it facilitates will contribute to reducing the burden on biologists by promoting the use and accuracy of knowledge discovery from computational genome comparison.

### References

ACT (accessed August 2004): **http://www.sanger.ac.uk/Software/ACT/**

Altschul SF, Gish W, Miller W, Meyers EW, Lipman DJ. 1990. Basic local alignment search tool. *J Mol Biol* **215**(3): 403–410.

Anjum MF, Lucchini S, Thompson A, Hinton JCD, Woodward MJ. 2003. Comparative genomic indexing reveals the phylogenomics of *Escherichia coli* pathogens. *Infect Immun* **71**(8): 4674–4683.

BioJava (accessed August 2004): **http://www.biojava.org**

Chapman B, Chang J. 2000. BioPython: Python tools for computational biology. *SIGBIO Newslett* **20**(2): 15–19.

Delcher AL, Kasif S, Fleischmann RD, *et al.* 1999a. Alignment of whole genomes. *Nucleic Acids Res* **27**(11): 2369–2376.

Delcher AL, Harmon D, Kasif S, White O, Salzberg SL. 1999b. Improved microbial gene identification with GLIMMER. *Nucleic Acids Res* **27**(23): 4636–4641.

Devedzic V. 2002. Understanding ontological engineering. *Commun ACM* **45**(4): 136–144.

Gennari J, Musen MA, Fergerson RW, *et al.* 2002. *The Evolution of Protégé: An Environment for Knowledge-based Systems Development*. Technical Report, Biomedical and Health Informatics, University of Washington, DC.

Haarslev V, Möller R. 2001. RACER system description. *Proceedings of the International Joint Conference on Automated Reasoning, IJCAR 2001*, Goré R, Leitsch A, Nipkow T (eds). Springer–Verlag: Berlin; 701–705.

Haarslev V, Möller R. 2003. Description logic systems with concrete domains: applications for the semantic web. In *Proceedings of the International Workshop on Knowledge Representation Meets Databases (KRDB-2003). Hamburg, Germany*. Technical University of Aachen.

Kurtz S, Schleiermacher C. 1999. REPuter: fast computation of maximal repeats in complete genomes. *Bioinformat Appl Note* **15**(5): 426–427.

McClelland M, Florea L, Sanderson K, *et al.* 2000. Comparison of the *Escherichia coli* K-12 genome with sampled genomes of *Klebsiella pneumoniae* and three *Salmonella enterica* serovars, Typhimurium, Typhi and Paratyphi. *Nucleic Acids Res* **28**(24): 4974–4986.

McGuinness DL, van Harmelen F. 2004. OWL web ontology language overview (**http://www.w3.org/TR/owl-features/**).

Microbase (accessed September 2004): **http://www.microbase.org.uk**

Mouse Genome Informatics (accessed August 2004): **http://www.informatics.jax.org/**

Parkhill J. 2002. The importance of complete genome sequences. *Trends Microbiol* **10**(5): 219–220.

Parkhill J, Achtman M, James KD, *et al.* 2000. Complete DNA sequence of a serogroup A strain of *Neisseria meningitidis* Z2491. *Nature* **404**: 502–506.

Pearson WR, Lipman DJ. 1988. Improved tools for biological sequence comparison. *Proc Natl Acad Sci USA* **85**: 2444–2448.

Schwartz S, Zhang Z, Frazer KA, *et al.* 2000. PipMaker — a web server for aligning two genomic DNA sequences. *Genome Res* **10**(4): 577–586.

Sequence Ontology (accessed August 2004): **http://song.sourceforge.net**

Smith TF, Waterman MS. 1981. Identification of common molecular subsequences. *J Mol Biol* **147**: 195–197.

Stajich JE, Block D, Boulez K, *et al.* 2002. The Bioperl toolkit: perl modules for the life sciences. *Genome Res* **12**: 1611–1618.

The Gene Ontology Consortium. 2000. Gene Ontology: tool for the unification of biology. *Nature Genet* **25**: 25–29.

Wexler Y, Yakhini Z, Kashi Y, Geiger D. 2004. Finding approximate tandem repeats in genomic sequences. In *Proceedings of the Eighth Annual International Conference on Computational Molecular Biology*. ACM Press: New York; 223–232.

Copyright © 2004 John Wiley & Sons, Ltd.
